# Beyond chronological age: maturity offset is positively associated with performance in throwing-related tests in youth track and field throwing athletes

**DOI:** 10.3389/fspor.2026.1765224

**Published:** 2026-03-18

**Authors:** Jannik Severin, Simon Nolte, Thomas Abel, Marlen Schapschröer

**Affiliations:** 1 Institute of Movement and Neurosciences, German Sport University, Cologne, Germany; 2 Institute of Exercise Training and Sport Informatics, German Sport University, Cologne, Germany

**Keywords:** performance interpretation, physical fitness, selection bias, talent identification, youth athletics

## Abstract

**Introduction:**

This study examined the influence of maturity offset, defined as distance from age at peak height velocity, on sport motor test performance in female and male youth track and field throwing athletes aged 13–19 years. The aim was to improve test interpretation and support valid talent identification.

**Methods:**

A retrospective data analysis was conducted on 468 observations (female: *n* = 252; male: *n* = 216) from 379 junior throwing athletes (female: *n* = 205, age: 15.5 ± 1.2 y; male: *n* = 174, age: 15.4 ± 1.1 y). Maturity offset was estimated with the widely used non-invasive Mirwald method. Performance was assessed using a standardized test battery including sprints, jumps, power-oriented throwing tests and a 12-minute run. Associations between maturity offset and test performance were analyzed using linear mixed-effects models.

**Results:**

Higher maturity offset was positively related to performance of throwing-related tests, corresponding to an increase of 12.8% (95% CI: 10.3, 15.3; *β* = 0.37) in the backward overhead shot throw and 11.9% (95% CI: 9.7, 14.1; *β* = 0.38) in the forward shot throw for athletes who had a maturity offset that was one year higher than their same-aged peers. Effects on other motor tests were small to trivial (−2.9 to 2.3% performance difference; *β* = −0.14 to 0.14).

**Discussion:**

Maturity offset affects physical test outcomes in youth throwers, especially in power-related throwing tasks. These findings highlight the importance of considering maturity offset when interpreting test results to reduce bias in talent identification and better support long-term athlete development.

## 1 Introduction

Youth sport talent identification represents a fundamental aspect of sports science in competitive sports, aiming to recognize young athletes with the highest levels of sport-specific potential and probability of excelling at the senior level (1). To support this goal, researchers and coaches have developed sport-specific test batteries that assess key physical and anthropometric characteristics (2) that influence talent selection decisions (3). Numerous studies have analyzed performance metrics of youth athletes across diverse sports (2, 4–12). In this context, a key challenge lies not only in the standardization and comparability of tests (13), but also in the interpretation of results, which must consider contextual and group-specific factors. Differences in sex (14), specific sport disciplines, and even playing positions in team sports (15) can significantly influence test outcomes. Moreover, the impact of relative age (difference in chronological age between individuals within the same age group) (16, 17) and biological maturation (7, 11) on test performance or team selection of youth athletes has been increasingly recognized. Notably, biological maturation appears to exert a stronger influence on test performance than relative age (18, 19). As talent identification frequently relies on competition or test performance, the reasons mentioned consequently influence this process.

Biological maturation refers to the progression of processes leading individuals toward their fully developed adult state (20). This progression varies widely among individuals due to genetic and environmental factors (20). Maturation-related differences appear not only between individuals but also between sexes, with females reaching their age at peak height velocity (APHV) earlier than males (21). Biological maturation is commonly assessed using skeletal age, secondary sexual characteristics, or predictive models based on anthropometric measurements (22). A widely used non-invasive method is the Mirwald equation (21), which estimates the maturity offset (MO) - defined as the time before or after the APHV - using standing height, sitting height, body weight and chronological age (CA). By subtracting MO from CA, APHV can be derived. This approach allows athletes to be characterized relative to their maturation status, with higher MO values indicating that an athlete is further past PHV (i.e., more mature), and lower or negative values indicating that an athlete is closer to or before PHV (i.e., less mature). Beyond its relevance during the growth spurt itself, the subsequent phase until full maturation is of particular interest in track and field, as it coincides with crucial stages of talent identification.

Maturation-related influences on test results complicate the identification of truly talented individuals, as early physical advantages may not necessarily translate into long-term success (23–25). Grouping athletes by maturation status allows for a more meaningful interpretation of test results, as physical attributes significantly affect those outcomes (18, 19, 26). Early-maturing athletes tend to exhibit advantages in height, muscle mass, and strength during adolescence, often leading to superior athletic performance (5) and overrepresentation in talent development programs (17, 27, 28). Although these differences mostly diminish (17) or even reverse in favor of late-maturing individuals once full maturation is reached (29, 30), the developmental trajectory from the growth spurt to full maturity, as well as the extent to which the timing of the growth spurt affects performance during this period, remain to be clarified. In track and field, talent identification is frequently concentrated within this age range, which further emphasizes the critical role of maturation-related differences in shaping opportunities and future success. This dynamic places late-maturing athletes at particular risk of being overlooked, thereby limiting their access to training opportunities and potentially impairing their long-term development (31, 32). This developmental bias may lead to two types of misjudgment: false positives, where athletes are selected due to temporary maturation-related advantages, and false negatives, where athletes with substantial long-term potential are overlooked. The latter is of particular concern, as insufficient access to talent-oriented training and support may lead to the premature dropout of highly promising athletes from the sport system. Consequently, understanding how maturation affects performance metrics across various age groups before, during and after the growth spurt is essential for refining talent identification and development strategies (22, 33).

In team sports, substantial evidence highlights the impact of maturation on sprint performance, strength, power, and sport-specific skills (7–11, 34–36). The various studies have examined both the direct impact of the growth spurt and the subsequent years. In contrast, data on track and field disciplines, particularly in throwing events, remain scarce. A recent study involving 54 male 13-year-old sprint and long jump athletes identified significant maturity-related differences in long jump and 60 m sprint performance, as well as in all physical testing variables (e.g., countermovement jump) (4). Similarly, maturity status was found to partially explain variance in 800 m performance in 89 male athletes aged 13–15 years (37).

Track and field's throwing events heavily rely on strength and power (38) which are influenced by anthropometric measures, such as body mass and height. While some studies suggest that throwers differ anthropometrically from peers in other events (39) even at younger ages (40) the specific influence of timing and status of maturation on their performance has not yet been sufficiently analyzed and understood (38). Additionally, most available studies focus on male athletes, resulting in underrepresentation of female populations and a significant gap in the current body of research (41, 42).

Although previous research has demonstrated that maturity status can significantly influence test performance in track and field athletes, it remains unclear to what extent these effects persist into older age groups. While several authors suggest that the influence of maturation diminishes as athletes mature (43, 44), little is known about the critical period after APHV. In particular, the mechanisms underlying performance changes in this transitional phase, as well as the potential long-term impact of maturation timing beyond the growth spurt, are not yet well understood. Addressing this gap is essential for accurately interpreting performance data and for developing appropriate testing and training strategies.

The German Athletics Association uses a comprehensive test battery to assess athletes competing at regional to national level (45). This dataset provides an opportunity to address the highlighted research gap by analyzing performance metrics in combination with MO data, while also bringing attention to the scarcely explored area of female athletes (41, 42). Therefore, this study aims to evaluate the influence of MO, while accounting for CA, on sport motor test results in female and male youth throwing athletes. By linking MO with test performance metrics, this study seeks to improve the interpretation of test results and contribute to more equitable and effective talent identification and development strategies in track and field throwing disciplines.

## 2 Material and methods

### 2.1 Study design

In this retrospective analysis, data collected by the German Athletics Association between 2016 and 2023 was analyzed. The test battery was typically conducted between September and November by regional and national athletics associations, involving athletes competing at the regional to national level. The dataset reflects real-world, multi-site routine testing under applied talent identification conditions conducted over several years. Therefore, minor measurement variability across locations, equipment, and test occasions cannot be fully excluded. To minimize such variability, testing procedures and measurement equipment were standardized using a comprehensive test manual provided by the German Athletics Association (45), which was implemented by experienced instructors from regional and national associations. The test battery is typically conducted with athletes aged 14–16 years. This age range is particularly relevant to track and field, as it aligns with key talent identification processes within the German Athletics Association (45) and the transition from U16 to U18 competition levels, where athletes first compete internationally (e.g., European Athletics U18 Championships). Moreover, this period corresponds to the years immediately following APHV, when growth velocity has already slowed (44), providing an appropriate window to examine how maturation-related differences continue to affect performance beyond the growth spurt, while accounting for CA.

The study was reviewed by the Ethics Committee of the German Sport University Cologne and conducted in accordance with local legislation and institutional requirements. Written informed consent for participation was not required from the participants or their legal guardians because this retrospective study analyzed pseudonymized anthropometric and sport motor test data from youth track and field athletes, collected during routine assessments of the regional or national associations. All athletes (and/or their legal guardians) had provided informed consent for participation in the routine testing procedures. The study complied with key principles of the Declaration of Helsinki.

### 2.2 Participants

A total of 468 observations (female: *n* = 252; male: *n* = 216) from 379 junior throwing athletes (female: *n* = 205, age: 15.5 ± 1.2 y; male: *n* = 174, age: 15.4 ± 1.1 y), all competing at regional to national level, were included in the analyses. Of these athletes, 60 (female: *n* = 29; male: *n* = 31) were assessed twice, 13 (female: *n* = 9; male: *n* = 4) were assessed three times, and one male athlete was assessed four times within the data collection period. Discipline-specific information (shot put, discus, hammer, javelin) was not available in the dataset. Therefore, all athletes were analyzed as a single group.

### 2.3 Measurements

Anthropometric measures, including body mass (BM), body height (BH) and sitting height (SH) were obtained by experienced examiners. Before the sport motor tests, all participants completed a self-selected warm-up routine, with sufficient time provided for each participant to warm up as needed. Given their competition experience, participants were likely familiar with preparing appropriately for maximal efforts. The sport motor tests (45) comprised the following:

- 60 m sprint timed over multiple intervals (0–10 m, 10–30 m, 0–30 m, 30–60 m, 0–60 m): From a standing start with 1 m distance from the electric timekeeping
- Forward shot throw (FOST): Using a 3 kg shot for females and a 4 kg shot for males
- Backward overhead shot throw (BOST): Using a 3 kg shot for females and a 4 kg shot for males
- Triple hop for distance (TH): Performed with single leg and a 5 m approach, with the mean distance calculated from both legs
- Five-jump test for distance (FJT): Performed with alternating legs and a 10 m approach
- Countermovement jump (CMJ): explosively jumping vertically after squatting to a self-selected depth
- Drop jump (DJ): Conducted from a height of 30 cm, with efficiency calculated as flight time squared divided by ground contact time
- 12-Minute run test (12MR): distance covered within 12 min

Each athlete performed two attempts per test (except for the 12MR), with the better attempt selected for analysis. All tests were conducted on the same day, with adequate time for recovery between attempts and tests. Testing was carried out in indoor athletics halls with tartan surface, while the 12MR was performed outdoors on 400 m tracks. Due to the large number of participants, test order varied across athletes, whereas the 12MR was consistently conducted as the final test.

### 2.4 Maturity offset

The widely used (5, 7, 8, 28, 33, 34, 46) non-invasive method proposed by Mirwald et al. (21) was used to estimate the maturity offset (MO) which—despite its discussed potential limitations (47, 48)—remains the most commonly applied approach to assess biological maturation in youth athletes. This method applies sex-specific prediction equations that incorporate anthropometric measures, including BM, BH, and SH to calculate the time before or after APHV. Leg length, derived as the difference between BH and SH, along with chronological age at the time of measurement, are also included in the calculation. By subtracting MO from CA, the predicted APHV can be derived.

Boys: Maturity offset = −9.236 + 0.0002708 × (leg length × sitting height) - 0.001663 × (age × leg length) + 0.007216 × (age × sitting height) + 0.02292 × (weight/height × 100)

Girls: Maturity offset = −9.376 + 0.0001882 × (leg length × sitting height) + 0.0022 × (age × leg length) + 0.005841 × (age × sitting height) - 0.002658 × (age × weight) + 0.07693 × (weight/height × 100).

### 2.5 Statistical analysis

Descriptive statistics and exploratory visualizations were used to examine the relationships between CA, MO, and sport motor test performances. Maturity was quantified using the predicted APHV. To obtain a cohort-specific and sex-adjusted maturity indicator, we calculated the sex-specific mean APHV within our sample and expressed each observation as the difference of the individual predicted APHV from this sex-specific mean APHV. This variable (dMO) therefore reflects whether an athlete is earlier or later maturing relative to same-sex peers, and was included as the main predictor of interest in the subsequent analyses.

For each motor test variable, a linear mixed-effects model (LMM) was fitted with test performance as the dependent variable. LMMs were chosen because they allow to model hierarchical data with different numbers of observations per subject without excluding data, in contrast to more traditional methods such as ANOVA. Known predictors of youth sport performance (CA, sex, and their interaction) were included in the model as fixed effects. The variable of interest, dMO, was added as an additional fixed effect. Participant ID was modeled as a random intercept to account for repeated measures. The full model notation was: performance∼CA * Sex+dMO+(1 | id).

Model coefficients and likelihood profile based 95% confidence intervals were extracted. To allow for comparison across tests with different scales, estimates were standardized relative to the mean performance of each test (as percentage over/underperformance). Several authors have recommended expressing effect sizes in a practically meaningful form, such as percentage changes, to facilitate interpretation in applied contexts (49, 50). In addition, the standardized beta coefficient was reported as a classical effect size to provide a conventional reference alongside the percentage-based over/underperformance. Following common interpretations, standardized beta coefficients of ≥0.1, ≥0.3, and ≥0.5 can be considered to represent small, medium, and large effects, respectively (51). For tests in which lower values indicated better performance (e.g., sprint times), coefficients were multiplied by −1 to ensure consistent interpretation across measures.

All analyses were conducted in R (Version 4.5.0). Linear mixed-effects models were fitted with the *lme4* package (Version 1.1.37) (52). The analysis code and data are available here https://doi.org/10.5281/zenodo.17591244.

## 3 Results

A total of 468 observations from 379 athletes were analyzed. Descriptive statistics (means and standard deviations) are presented in Table 1.

**Table 1:** Descriptive statistics of female and male throwing athletes.

	Female		Male	
Variable	n	Mean ± SD	n	Mean ± SD
BM, kg	252	67.7 ± 9.7	216	79.5 ± 12.6
BH, cm	252	172.1 ± 6.2	216	183.9 ± 7.5
SH, cm	252	90.7 ± 3.7	216	96.1 ± 4.4
CA, years	252	15.5 ± 1.2	216	15.4 ± 1.1
APHV, years	252	12.3 ± 0.6	216	12.9 ± 0.6
MO, years	252	3.2 ± 0.9	216	2.5 ± 1.1
0–10 m, s	202	1.88 ± 0.09	160	1.74 ± 0.10
10–30 m, s	202	2.74 ± 0.15	160	2.48 ± 0.16
0–30 m, s	235	4.61 ± 0.21	200	4.23 ± 0.24
30–60 m, s	242	3.99 ± 0.26	204	3.55 ± 0.28
0–60 m, s	242	8.60 ± 0.46	209	7.78 ± 0.50
FOST, m	249	12.16 ± 1.68	212	14.58 ± 2.36
BOST, m	233	13.01 ± 2.06	195	15.69 ± 2.77
TH, m	208	7.38 ± 0.95	168	8.75 ± 1.01
FJT, m	163	13.46 ± 1.15	140	15.25 ± 1.40
CMJ, cm	207	35.53 ± 4.76	173	47.09 ± 6.68
DJ	237	1.44 ± 0.34	204	1.57 ± 0.38
12MR, m	207	2,351 ± 225	180	2,571 ± 271

BM, body mass; BH, body height; SH, sitting height; CA, chronological age; APHV, age at peak height velocity; MO, maturity offset; FOST, forward shot throw; BOST, backward overhead shot throw; TH, triple hop for distance; FJT, five-jump test for distance; CMJ, countermovement jump; DJ, drop jump; 12MR, 12-minute run test.

Visual inspection indicated a linear relationship between CA and both, MO and performance in all motor tests. For both sexes, MO increased with increasing CA, with female athletes showing higher MO compared to male athletes of the same CA. The rate of performance increase per year of CA differed between males and females, with males showing steeper slopes. These differences were accounted for in the statistical models by including the interaction between CA and sex.

### 3.1 Linear mixed-effects model

The linear mixed-effects models revealed an influence of MO on different test performance metrics. Visual inspection and exploratory analyses provided no evidence for sex-specific effects of MO in the present dataset. Therefore, the effects described below apply to athletes of the same sex and chronological age. MO was positively related to test performance of throwing-specific tests, corresponding to an increase of 12.8% (95% CI: 10.3, 15.3) for BOST and 11.9% (95% CI: 9.7, 14.1) for FOST per additional year of MO. This overperformance corresponds to a standardized beta coefficient of 0.37 for BOST and 0.38 for FOST, which represents a medium effect. The 12MR (−2.8%, 95% CI: −5.0, −0.6; *β* = −0.14) and DJ (−2.9%, 95% CI: −7.6, 1.8; *β* = −0.07) showed negative associations with MO. The standardized effects were small (12MR) to trivial (DJ), and the DJ 95% CI included zero. For the remaining test variables, estimated performance changes per additional year of MO were in a range from 0.7% (95% CI: −0.5, 1.9) for 0–10 m to 2.3% (95% CI: 0.9, 3.7) for 30–60 m. Among these, MO showed a small effect (*β* = 0.11–0.14) for FJT (2.1%, 95% CI: 0.1, 4.1), 10–30 m (1.4%, 95% CI: 0.1, 2.7), 0–60 m (1.9%, 95% CI: 0.8, 3.0) and 30–60 m.

**Figure 1:**
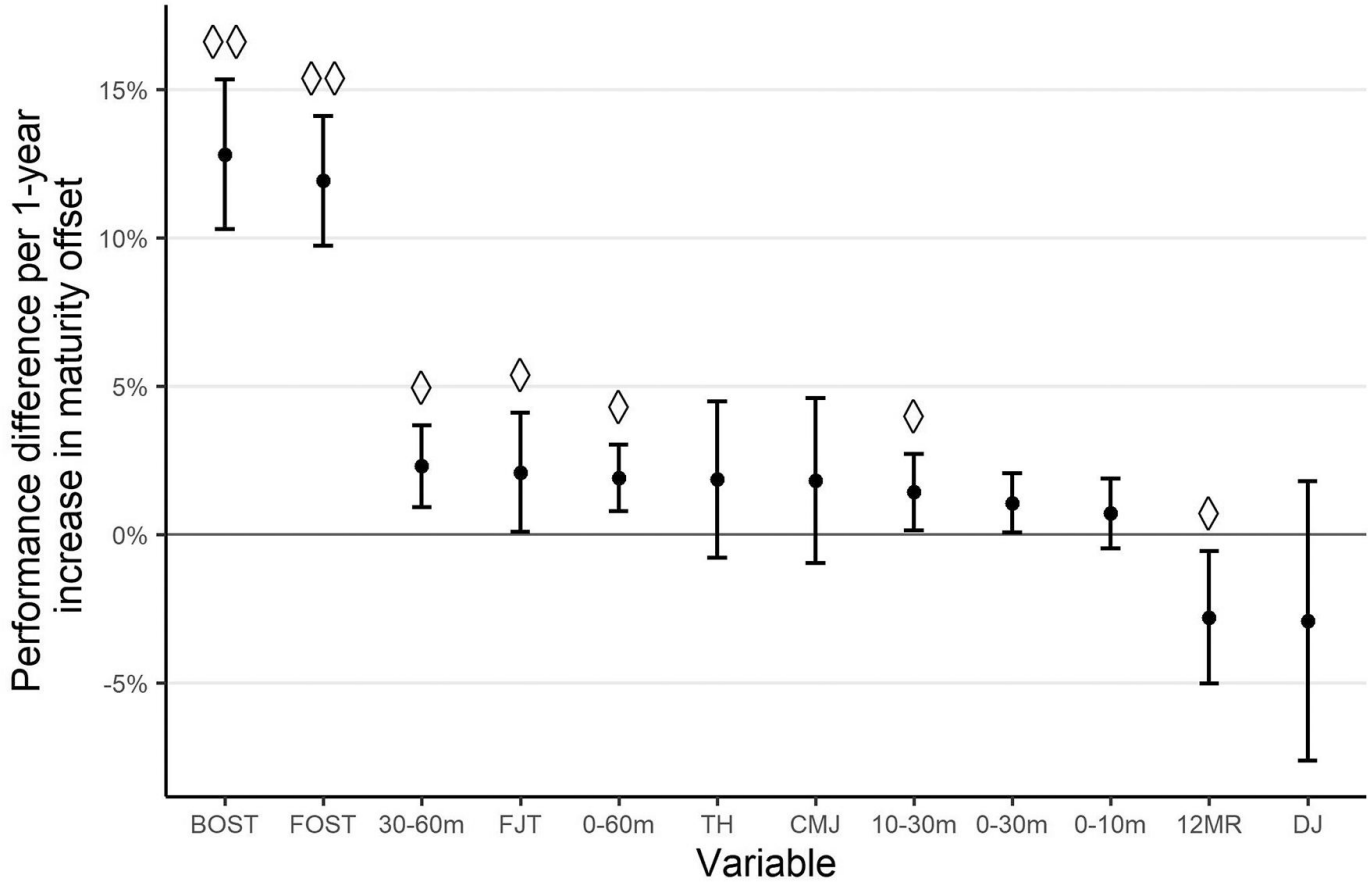
Relative overperformance for an athlete with a maturity offset that is one year higher compared to peers of the same chronological age and sex. Standardized beta coefficients are indicated as: ◊ ≥ 0.1, ◊◊ ≥ 0.3. BOST, backward overhead shot throw; FOST, forward shot throw; FJT, five-jump test for distance; TH, triple hop for distance; CMJ, countermovement jump; 12MR, 12-minute run test; DJ, drop jump.

A full overview of effect estimates is provided in Figure 1 and the Supplementary Material.

## 4 Discussion

This study examined the influence of MO, defined as years from APHV and adjusted for CA and sex, on sport motor test performance in male and female youth throwing athletes. The results indicated that MO was particularly associated with higher performance in throwing-specific tests.

To our knowledge, no previous study has specifically examined the influence of MO on sport motor test performance in youth throwing athletes after APHV. The analyzed age range is particularly relevant in track and field, as it aligns with key talent identification processes and the transition from U16 to U18 competition levels, where athletes begin to compete internationally (e.g., European Athletics U18 Championships). Understanding how MO affects test performance beyond the growth spurt may therefore contribute to more effective athlete identification and development strategies, since test results may influence talent selection decisions (3, 12).

Throwing-specific tests (BOST and FOST) demonstrated medium effect sizes based on conventional effect size thresholds (51). This corresponds to a performance advantage of 12.8% for BOST and 11.9% for FOST for each additional year of MO, indicating a practically meaningful positive impact of MO on throwing-related tests. In other words, an athlete who had a MO that was one year higher compared to counterparts of the same chronological age and sex overperformed them by 12.8% in BOST and 11.9% in FOST. These findings provide further evidence that MO substantially influences power-related performance outcomes in youth throwers and should therefore be considered when interpreting test results. Given prior evidence of maturity-related improvements in upper- and lower-body power in male basketball athletes (28), the present study expands the existing body of knowledge to female and male youth throwing athletes after the APHV and points to the development of strength and power capacities associated with advancing MO. At the same time, throwing performance is also influenced by anthropometric characteristics (e.g., body height and body mass) (38). However, available evidence suggests that strength- and power-generating capacities (and related lean mass) are more strongly associated with throwing performance than body mass *per se* (53). The present findings in male athletes extend those of Zhao & Zhao (38), who revealed pronounced age-related physical development in male throwing athletes aged 14–18. In contrast to the findings of this study, the same authors did not observe differences in female throwing athletes for BOST and FOST when grouping by age, although both tests were significantly correlated with throwing performance. Taken together, the findings of this study underline the importance of considering MO when interpreting power-oriented test results in youth throwers. Without accounting for MO, there is a risk of overinterpreting the performance of athletes with higher MO or, conversely, overlooking athletes with lower MO who may have high potential. Under certain circumstances, athletes with lower MO should therefore be considered for special support (e.g., specific training or inclusion in a squad) despite their temporarily lower performance. This is particularly relevant given that these tests have been shown to differentiate between squad levels in youth track and field throwing athletes (12).

The CMJ was only marginally (1.8%, *β* < 0.1) influenced by MO, despite maturity-related differences being well-documented in other sports (8, 34, 42, 54). This may be explained by the bodyweight-dependent nature of the task, where gains in body mass counterbalance potential advantages in absolute power, highlighting that relative rather than absolute power is the critical factor for performance. While Arede et al. (28) observed maturity-related differences in absolute CMJ power output in basketball players, the absence of force-time data in the present study limits further interpretation. Similarly, the more technically demanding jumps (FJT: 2.1%, *β* = 0.11; TH: 1.9%, *β* < 0.1) showed only marginal associations with MO, in contrast to maturity related findings in handball (34, 46, 55). The technical complexity of these tasks, combined with the higher body mass typical for throwers (40), may explain these differences.

The influence of MO on sprint performance ranged from 0.7 to 2.3%, with small standardized beta coefficients observed for 10–30 m, 0–60 m and 30–60 m. However, given the small effect sizes and percentage differences, it remains questionable whether this has a meaningful impact on the interpretation of test results or on subsequent talent identification decisions. The results add to the findings of Zhao & Zhao (38), who found no significant differences in sprint results between chronological age groups in male and female athletes. While other studies reported maturity-related sprint differences across several sports and maturity stages (4, 7, 8, 34), these effects appear to diminish after the growth spurt. The anthropometric profile of throwers, including increased body mass (40), may contribute to this pattern given the close link between relative power output and sprint performance (56).

A higher maturity offset was negatively associated with 12MR performance (−2.8%, 95% CI: −5.0, −0.6), with a small beta coefficient (*β* = −0.14). This aligns with previous findings reporting better shuttle-run performance in later-maturing youth soccer players (57). Several mechanisms may explain this pattern in youth throwers after APHV. Training after APHV typically shifts toward strength- and technique-oriented content, often with comparatively less emphasis on aerobic endurance development. Moreover, running-based endurance training may be intentionally limited, given evidence for potential interference effects when resistance training is combined with excessive running (58). In addition, more advanced maturation is commonly accompanied by increases in body mass (22), which can disadvantage athletes in weight-bearing endurance field tests such as the 12MR.

Maturity offset showed a negative association with DJ performance (−2.9%), with a trivial standardized effect (*β* = −0.07). The corresponding confidence interval was wide and included zero (95% CI: −7.6, 1.8), indicating an imprecise estimate that should be interpreted with caution. One possible explanation is that the DJ is a technically demanding task requiring coordination and lower-limb stiffness, which can lead to substantial between-athlete variability and may be influenced by differing plyometric exposure. Moreover, increases in body mass with maturation (22) may affect reactive jump characteristics. Despite the uncertainty of the present estimate, the DJ reflects a substantial portion of the athletic requirements in track and field (59). Therefore, continued monitoring and development of DJ performance seems to be reasonable.

The findings highlight the relevance of MO in talent identification even after APHV, supporting the argument that it may have a meaningful influence on selection outcomes in addition to other factors like relative age (17, 18, 26). The strong associations between MO and throwing-specific tests suggest that athletes with advanced MO may be overrepresented in talent pathways, particularly in power-based disciplines like throwing. These results emphasize the need to interpret motor test outcomes within the specific context of sport and discipline (12, 15) to avoid biases in selection processes.

Unlike many previous studies that included only male participants, the present research analyzed both male and female throwers. The results indicated that the observed effects apply for both sexes, contributing to addressing the underrepresentation of female athletes in studies of maturational effects (42) and supporting more equitable approaches to athlete identification and development.

### 4.1 Practical implications

The findings of this study underscore the need to account for MO in the identification and development of youth throwing athletes. Since MO influences sport motor test performance, selection processes that rely solely on chronological age risk favoring early-maturing individuals while underestimating the potential of later-maturing peers. Coaches and sport scientists are therefore encouraged to integrate maturity assessments into both talent identification and training planning, even beyond the period of rapid growth. Tailoring talent identification processes and training programs to individual maturity profiles could help promote more equitable development pathways and optimize long-term athlete success.

### 4.2 Limitations and future directions

Since the data were collected as part of routine testing across multiple sites and over several years, minor measurement variability across locations, equipment, and test occasions may have occurred. Also, given the absence of event classification (shot put, discus, hammer, javelin), the present findings should be interpreted as reflecting general characteristics of junior throwing athletes within the German Athletics Association test program rather than discipline-specific profiles. Nevertheless, the dataset provides a rare opportunity to examine a large, practice-based sample over an extended period and may therefore offer insights into performance characteristics under applied talent identification conditions that complement findings from more tightly controlled laboratory or single-site studies.

The estimated APHV values in this study (female: 12.3 ± 0.6 years; male: 12.9 ± 0.6 years) were somewhat earlier than reported in comparable cohorts (22), particularly in male athletes. This likely reflects a strong selection bias, which is widespread in competitive sports (32, 60, 61). Late-maturing athletes are often underrepresented at higher performance levels. Consequently, only a small proportion of late-maturing athletes typically progresses into senior categories, which may limit the generalizability of our findings across the full spectrum of maturation and highlights the need for further research. Furthermore, throwing athletes possess distinct anthropometric characteristics that limit the generalizability of findings to other disciplines (40). Given the strong influence of anthropometry on performance (38), discipline-specific research remains essential.

The Mirwald method (21), while non-invasive and widely used, provides an indirect estimation of maturity offset and, as with any estimation, is less precise than direct assessments. As the prediction equation was derived from a cohort with different anthropometric characteristics than youth throwing athletes, such differences in anthropometry may affect the accuracy of the estimated maturity offset. The results have to be interpreted with caution, as potential discrepancies between estimated and actual maturity offset, especially with increasing CA, potentially impacts the data (47, 48, 62).

The cross-sectional design restricts causal interpretations and limits conclusions regarding long-term developmental effects. Future studies should employ longitudinal approaches to capture how maturational timing influences performance trajectories and career progression. Finally, future research should continue to address the lack of data on female youth throwers to ensure that talent development strategies are inclusive and evidence-based for both sexes.

### 4.3 Conclusion

This study demonstrates that maturity offset substantially influences sport motor test performance, particularly throwing-specific assessments, in youth athletes after the APHV. By addressing the underrepresentation of female athletes, it adds to the limited evidence base on sex-specific developmental patterns in throwing disciplines. Incorporating maturity-related information into test interpretation and selection decisions may help reduce bias, provide fairer development opportunities, and enhance the long-term success of female and male youth track and field throwing athletes.

## Data Availability

The original contributions presented in the study are included in the article/Supplementary Material, further inquiries can be directed to the corresponding author. The analysis code and data are available here https://doi.org/10.5281/zenodo.17591244.
