# Supplementary material for: Beyond chronological age: maturity offset is positively associated with performance in throwing-related tests in youth track and field throwing athletes
Source: Front Sports Act Living. 2026 Mar 18;8:1765224. doi: 10.3389/fspor.2026.1765224 (PMC13040386; doi:10.3389/fspor.2026.1765224)
Supplement: Supplementary file 1 [file Table1.docx]

Supplementary Material

# Supplementary Table

Table 2. Relative overperformance and classic effect size for an athlete with a maturity offset that is 1 year higher compared to counterparts of the same chronological age and sex.

| **Variable** |  | **Overperformance in %** |  | **Lower CI** | **Upper CI** |  | **β** |
| --- | --- | --- | --- | --- | --- | --- | --- |
| BOST |  | 12.8 |  | 10.3 | 15.3 |  | 0.37 |
| FOST |  | 11.9 |  | 9.7 | 14.1 |  | 0.38 |
| 30-60m |  | 2.3 |  | 0.9 | 3.7 |  | 0.14 |
| FJT |  | 2.1 |  | 0.1 | 4.1 |  | 0.11 |
| 0-60m |  | 1.9 |  | 0.8 | 3.0 |  | 0.14 |
| TH |  | 1.9 |  | -0.8 | 4.5 |  | 0.07 |
| CMJ |  | 1.8 |  | -1.0 | 4.6 |  | 0.05 |
| 10-30m |  | 1.4 |  | 0.1 | 2.7 |  | 0.11 |
| 0-30m |  | 1.1 |  | 0.1 | 2.1 |  | 0.09 |
| 0-10m |  | 0.7 |  | -0.5 | 1.9 |  | 0.06 |
| 12MR |  | -2.8 |  | -5.0 | -0.6 |  | -0.14 |
| DJ |  | -2.9 |  | -7.6 | 1.8 |  | -0.07 |

Abbreviations: BOST, backward overhead shot throw; FOST, forward shot throw; FJT, five-jump test for distance; TH, triple hop for distance; CMJ, countermovement jump; 12MR, 12-minute run test; DJ, drop jump; β, standardized beta coefficient.
